# Likely Correlation between Sources of Information and Acceptability of A/H1N1 Swine-Origin Influenza Virus Vaccine in Marseille, France

**DOI:** 10.1371/journal.pone.0011292

**Published:** 2010-06-25

**Authors:** Antoine Nougairède, Jean-Christophe Lagier, Laetitia Ninove, Catherine Sartor, Sékéné Badiaga, Elizabeth Botelho, Philippe Brouqui, Christine Zandotti, Xavier De Lamballerie, Bernard La Scola, Michel Drancourt, Ernest A. Gould, Rémi N. Charrel, Didier Raoult

**Affiliations:** 1 Fédération de Microbiologie, Hôpital de la Timone, Assistance Publique-Hôpitaux de Marseille, Marseille, France; 2 UMR 190 Pathologies Virales Emergentes, Institut de Recherche pour le Développement-Université de la Méditerranée, Marseille, France; 3 Unité de Recherche sur les Maladies Infectieuses et Tropicales Emergentes UMR CNRS 6236 IRD 3R198, Université de la Méditerranée, Marseille, France; 4 Comité de Lutte contre les Infections Nosocomiales. Hôpital de la Conception, Assistance Publique-Hôpitaux de Marseille, Marseille, France; 5 Service d'Accueil des Urgences. Hôpital Nord, Assistance Publique-Hôpitaux de Marseille, Marseille, France; 6 Service des Maladies Infectieuses et Tropicales. Hôpital Nord, Assistance Publique-Hôpitaux de Marseille, France; University of Medicine & Dentistry of New Jersey-New Jersey Medical School, United States of America

## Abstract

**Background:**

In France, there was a reluctance to accept vaccination against the A/H1N1 pandemic influenza virus despite government recommendation and investment in the vaccine programme.

**Methods and Findings:**

We examined the willingness of different populations to accept A/H1N1vaccination (i) in a French hospital among 3315 employees immunized either by in-house medical personnel or mobile teams of MDs and (ii) in a shelter housing 250 homeless persons. Google was used to assess the volume of enquiries concerning incidence of influenza. We analyzed the information on vaccination provided by Google, the website of the major French newspapers, and PubMed. Two trust Surveys were used to assess public opinion on the trustworthiness of people in different professions. Paramedics were significantly more reluctant to accept immunisation than qualified medical staff. Acceptance was significantly increased when recommended directly by MDs. Anecdotal cases of directly observed severe infections were followed by enhanced acceptance of paramedical staff. Scientific literature was significantly more in favour of vaccination than Google and French newspaper websites. In the case of the newspaper websites, information correlated with their recognised political reputations, although they would presumably claim independence from political bias. The Trust Surveys showed that politicians were highly distrusted in contrast with doctors and pharmacists who were considered much more trustworthy.

**Conclusions:**

The low uptake of the vaccine could reflect failure to convey high quality medical information and advice relating to the benefits of being vaccinated. We believe that the media and internet contributed to this problem by raising concerns within the general population and that failure to involve GPs in the control programme may have been a mistake. GPs are highly regarded by the public and can provide face-to-face professional advice and information. The top-down strategy of vaccine programme management and information delivered by the Ministry of Health could have aggravated the problem, because the general population does not always trust politicians.

## Introduction

Following the confirmation by the World Health Organization, that A/H1N1 influenza virus had reached pandemic proportions; rapid implementation of large-scale immunization programmes was considered essential to reduce the burden of disease. This perception reflected previous evidence based on the experience of seasonal influenza [1], [2], [3], [4], [5]. Moreover, it has been demonstrated that influenza vaccination of widely different human categories, for example, healthy adults of all ages [3], children [5] or high risk populations such as the elderly [1], [4] may have a significant impact on hospitalization rate, influenza associated mortality, global morbidity and mortality. In support of this argument, rapid isolation of the novel A/H1N1 influenza virus in North America inevitably led to the rapid development of vaccine production after the first cases were reported [6].

In France, the Ministry of Health took the decision to purchase 94 million doses of vaccine with which to provide the capacity to organize an immunization programme targeting the entire French population (65 million inhabitants). Pandemic management, immunization protocols and logistics utilised a top-down strategy. Within the population, different groups were identified by the Ministry of Health, amongst which Health care workers (HCWs), from the public hospital system and private clinics, were ranked at the highest priority [7]. They were immunized at their workplace by trained personnel deployed as an occupational medical unit (OMU). Children were similarly provided with immunization in their schools. The remaining French population, including HCWs such as private nurses and practitioners, were offered vaccination at centres created in gymnasia, public buildings and other readily accessible sites [8]. Surprisingly, general practitioners (GPs) were not included in the execution of any of these protocols and therefore were unable to provide a coordinated contribution to the immunization efforts. Vaccination began on October 20^th^, November 12^th^, and November 25^th^, 2009 for public hospital HCWs, the general population, and the education system (except teachers who were included in the vaccination programme dedicated to the general population rather than the educational cohort), respectively. It is important to note that the safety of the vaccine and especially of the Squalene adjuvant was debated, relayed and continuously questioned by the French media. Opinion polls reported widespread suspicion of A/H1N1 vaccines and showed that even among HCWs, only a low proportion considered A/H1N1 vaccination favourably. We believe that as a result of this, initial vaccination rates remained low: by November 24^th^, only 18% (140,000/800,000) of the HCWs had been immunized during the first five weeks; moreover, only 460,000 doses had been administrated to the general population, representing 3.5% of the 13 million persons initially eligible [9].

Our hypothesis is that the acceptability of the vaccine in any French group of people is highly influenced by the source, and resulting nature, of information available. Here, using immunisation rates, we have analysed the level of acceptability of the A/H1N1 vaccine in a French public hospital amongst HCWs and also in a shelter for homeless persons located in Marseille. We also compared the volume of Google enquiries relating to influenza and bronchiolitis, with local epidemiological data. Finally, we compiled and analysed trends of A/H1N1 vaccination perception in (i) medical sources (PubMed), used primarily by medical and scientific staff, and (ii) Google and newspaper websites.

## Materials and Methods

### A/H1N1 vaccination campaign in La Conception hospital

The La Conception public hospital (LCPH) located in Marseille, (France) comprises a total of 618 beds: 352 medical beds (including 20 adult and 15 neonatal intensive care beds), 167 surgical beds, 99 gynaecological and obstetric beds and an emergency department, operating 24 hours a day. The number of hospital employees was obtained from the administration records. The 3315 employees, comprise 774 medical workers (medical residents and students), 1927 paramedics, 395 technical employees and 219 administrative staff. On October 22^nd^, 2009, we introduced the A/H1N1 vaccination programme to LCPH. Hospital employees were invited to attend the occupational medicine unit to receive the vaccine. On November 2^nd^, 2009, the vaccination policy was reinforced by introducing a mobile vaccination facility (MVF) operated by the infection control committee [10]; influenza vaccination of hospital employees was thus accomplished by delivering the vaccine directly to the patient care units by the physicians who informed HCWs of the availability and advisability of A/H1N1 vaccination. Over a period of 6 weeks, data were collected, anonymised and analysed in terms of time and occupational distribution. Following national regulations, this procedure did not require a specific consent from HCWs. In addition, all specific events relating to the pandemic during this period which concerned the LCPH directly (such as patient infected in intensive care unit), were recorded.

### A/H1N1 vaccination of the homeless population in Marseille

On December 20th, 2009, an A/H1N1 vaccination programme was organized in a shelter for homeless persons located in Marseille [11] which can accommodate about 300 people each night. It was approved by the DDASS (the French sanitary and social agency) which provided the vaccine. The procedure consisted of (i) a medical interview, (ii) the signature of an informed written consent form by each individual, and (iii) the delivery of a certificate of vaccination. The medical staff, including 8 MDs, 2 medical students and a social worker, visited the shelter once. All homeless individuals present at the time were informed about the presence of a medical team and the availability of the vaccine; they then decided whether or not to meet with a doctor. During the interview of those that agreed to meet the doctor, 3 questions were asked regarding flu vaccination: (1) Have you received a seasonal flu vaccine this year or the previous year? (2) Have you received the vaccine against A/H1N1 Influenza virus recently? (3) Have you heard about the flu vaccine? The gender of each person and the answers were recorded anonymously.

### Google enquiries on respiratory tract infections

We used Google Insight for Search (GIFS: http://www.google.com/insights/search/#)[12] to assess the number of Google enquiries. Data containing the search terms “grippe” (influenza in French) or “VRS + bronchiolite” (RSV + bronchiolitis in French) were obtained from 2005 with GIFS using a filter for the location (Provence-Alpes-Cote d'Azur (PACA), a region in south-eastern France representing approximately 8 million inhabitants). In addition, data relating to the relative number of enquiries containing the search terms “grippe” and “grippe – vaccination – vaccine” (the symbol “–” means without) were obtained from January 2009 with GIFS using a filter for the location (France).

Laboratory data from 2005 were extracted using the laboratory informatics system: Influenza and RSV analysis informatics codes were used to retrieve all nasopharyngeal aspirates and nasal swabs were tested. Viruses were detected using standard rapid immuno-chromatographic tests or a standard direct immunofluorescence technique. Following national regulations under the terms of Biomedical Research (Huriet-Sérusclat law # 881138), we were not required to obtain specific consent from patients (their signature at the hospital admission office warrants that all samples taken during hospitalization for diagnostic purpose are accessible for research). Data were anonymised and the number of positive samples was analysed by time distribution. From early April, A/H1N1 influenza virus was detected using two qRT-PCR assays [13]. The same procedure using the laboratory informatics system was applied to find the number of positive samples by time distribution. Data using immuno-chromatographic tests and direct immunofluorescence were merged with data using qRT-PCR assays (duplicates were eliminated).

### Comparison of information sources regarding influenza vaccination

On December 1^st^, 2009, an internet enquiry was undertaken with the search terms “grippe vaccination” (influenza vaccination in French) using either Google France (www.google.fr), or the query engine in the websites of 8 major French newspapers (see below) and “influenza vaccination” using PubMed (www.ncbi.nlm.nih.gov/pubmed/).

The first thirty pages or articles, or all issues of newspaper websites from April 2009 were classified into three categories by two separate examiners:

- 1: In favour of influenza vaccination: the page explains clearly that vaccination is the better option for fighting Influenza epidemics or pandemics.
- 2: Against influenza vaccination: the page gives a negative image of influenza vaccination (“a novel adverse effect following A/H1N1 vaccination”, “physicians continue to be suspicious about the A/H1N1 vaccination”, “the safety of vaccine questioned”, *etc*.).
- 3: Neutral regarding the influenza vaccination: the page relates facts without opinion.

Eight newspapers were investigated using their internet website. They were subclassified into pro- or con-government: pro-newspapers were Le Point, L'Express, Le Figaro and La Tribune reputed to have a political orientation in favour of the current government (conservative tendency); con-newspapers were Libération, Le Monde, L'humanité and Marianne reputed to have a political orientation divergent from that of the current government (socialist tendency).

### Trust surveys

We used two surveys (Trust Survey), one published by the Reader's Digest [14], and one organised by TNS Sofres poll organism [15]. Briefly, the first one was a consumer survey conducted during August–December 2008 involving 23,000 people in 16 European countries. The survey's primary objective was to find out which brands of goods Europeans trust the most in a range of consumer product categories. In addition, participants estimated their relative trust of individuals employed in 20 different professions. The second one was performed on December 8–9^th^, 2009, at the instigation of “Le Nouvel Observateur” (a French information magazine published weekly) on a French panel of 1000 persons representative of the population over 18 years-old, using the quotas method stratified according to the region in which they lived and the urban category. Participants estimated the prestige and utility of 25 professions.

## Results

### A/H1N1 vaccination programme in La Conception hospital

**Figure 1:**
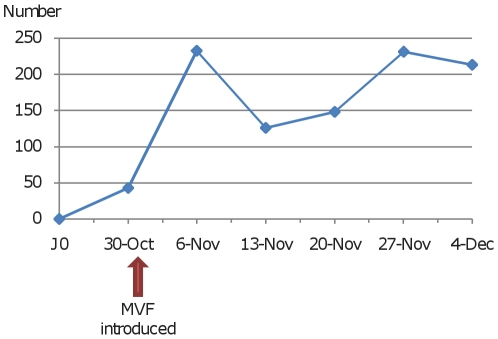
Time distribution of the 994 HCWs vaccinated in the LCPH.

**Figure 2:**
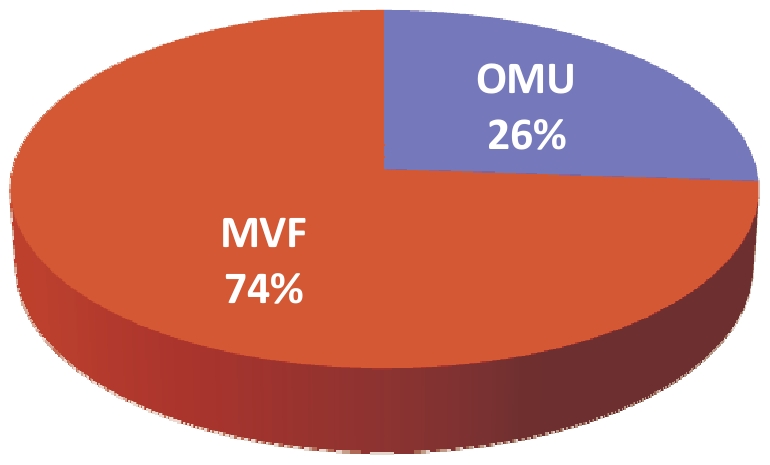
Distribution of vaccination uptake for the 994 HCWs vaccinated in the LCPH. OMU: Occupational medicine unit. MVF: Mobile vaccination facility.

**Figure 3:**
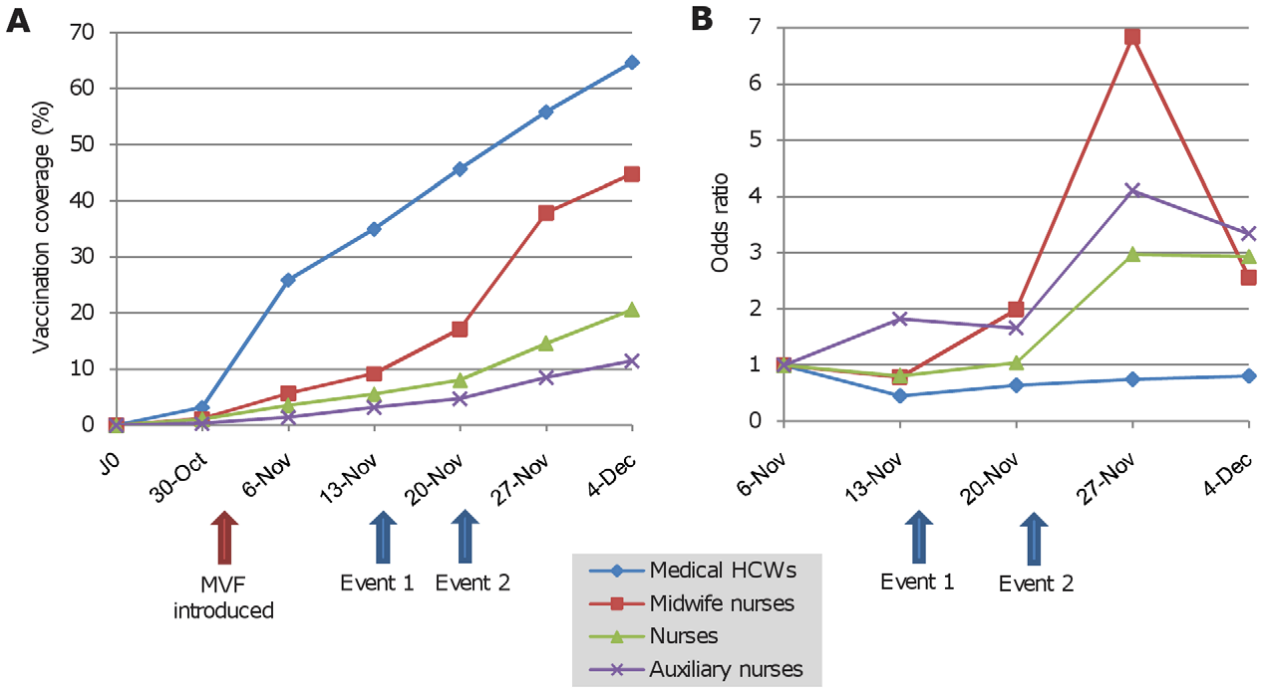
Time distribution of the vaccination by HCWs categories in the LCPH. Figure 3A indicates the vaccination coverage and figure 3B indicates the probability of being immunized (chi square for trend; the period from October 31^st^ to November 6^th^ was considered as baseline). Event 1: First patient admitted in ICU. Event 2: Two pregnant women admitted in ICU. *p-value* (chi square for trend test): 0.30, <0.001, <0.0001 and <0.01 for medical HCWs, midwife nurses, nurses and auxiliary nurses respectively.

Over a period of 6 weeks, 998 employees (30.1%) within LCPH were immunized against the novel A/H1N1 virus. Before the MVF was deployed, only 43 HCWs were immunized. However, when the two strategies (*ie* OMU and MVF) were deployed simultaneously, a minimum of 100 HCWs were vaccinated weekly (figure 1) resulting in a total of 260 (26.1%) HCWs vaccinated via the OCU and 738 (73.9%) vaccinated via the MVF. The relative risk of being vaccinated via the MVF among HCWs immunized was then 2.84 (95% IC: 2.54 to 3.17; chi square test: *p*<0.0001)(figure 2). Detailed analysis indicated that the vaccination coverage varied greatly according to the HCW category (table 1). Only 21.9% of paramedical HCWs chose to be immunized versus 64.5% of medical HCWs (chi square test: p<0.0001). The vaccination coverage remained low for paramedical HCWs until November 20^th^ and then increased suddenly, especially for midwife nurses. On the other hand, it increased progressively for medical HCWs (figure 3A). Importantly, the sudden increase for paramedical HCWs followed the admission of two pregnant women to the ICU which may have influenced the decision of these paramedical HCWs to be immunized (figure 3A and 3B): The relative risk of being vaccinated between November 21^st^ and December 4^th^, compared with the period from November 7^th^ to November 20^th^ was 2.68 (95% CI: 2.05 to 3.50; chi square test: *p*<0.0001) and 1.31 (95% CI: 1.09 to 1.58; chi square test: *p*<0.01) respectively, for the paramedical and medical HCWs.

**Table 1:** Vaccination in the LCPH among HCWs.

	Number	Vaccinated (%)	*p*-value* versus	
			(1)	(2)
**Medical staff (1)**	**774**	**499 (64.5%)**	–	–
**Paramedical staff (2)**	**1927**	**421 (21.9%)**	*<0.0001*	–
nurses	957	198 (20.7%)	*<0.0001*	–
midwife nurses	87	39 (44.8%)	*<0.001*	–
auxiliary nurses	506	58 (11.5%)	*<0.0001*	–
others	377	126 (33.4%)	*<0.0001*	–
**Technical employees**	**395**	**27 (6.8%)**	*<0.0001*	*0.69*
**Administrative employees**	**219**	**51 (23.3%)**	*<0.0001*	*<0.0001*

*: Chi square test: vaccinated versus unvaccinated.

### A/H1N1 vaccination programme amongst the homeless population in Marseille

In early December, an A/H1N1 vaccination programme was organized in a shelter for the homeless located in Marseille. Amongst the 250 homeless persons present in the shelter at this time, and which were proposed to receive information on A/H1N1 Influenza vaccine by the medical staff, 118 (47.2%) agreed to meet with a doctor. Of these, 109 (92.4%) were males one of whom had already been vaccinated and 117 agreed to be vaccinated after the medical interview. In total, 46.8% (117/250) of the 250 homeless persons were vaccinated during this one-day campaign (table 1). Whilst the majority (96.3%, 103/107) of homeless persons had heard about the pandemic flu vaccine, only 14.4% (13/90) had received a seasonal flu vaccine either during this year or the previous year.

### Internet-related information

**Figure 4:**
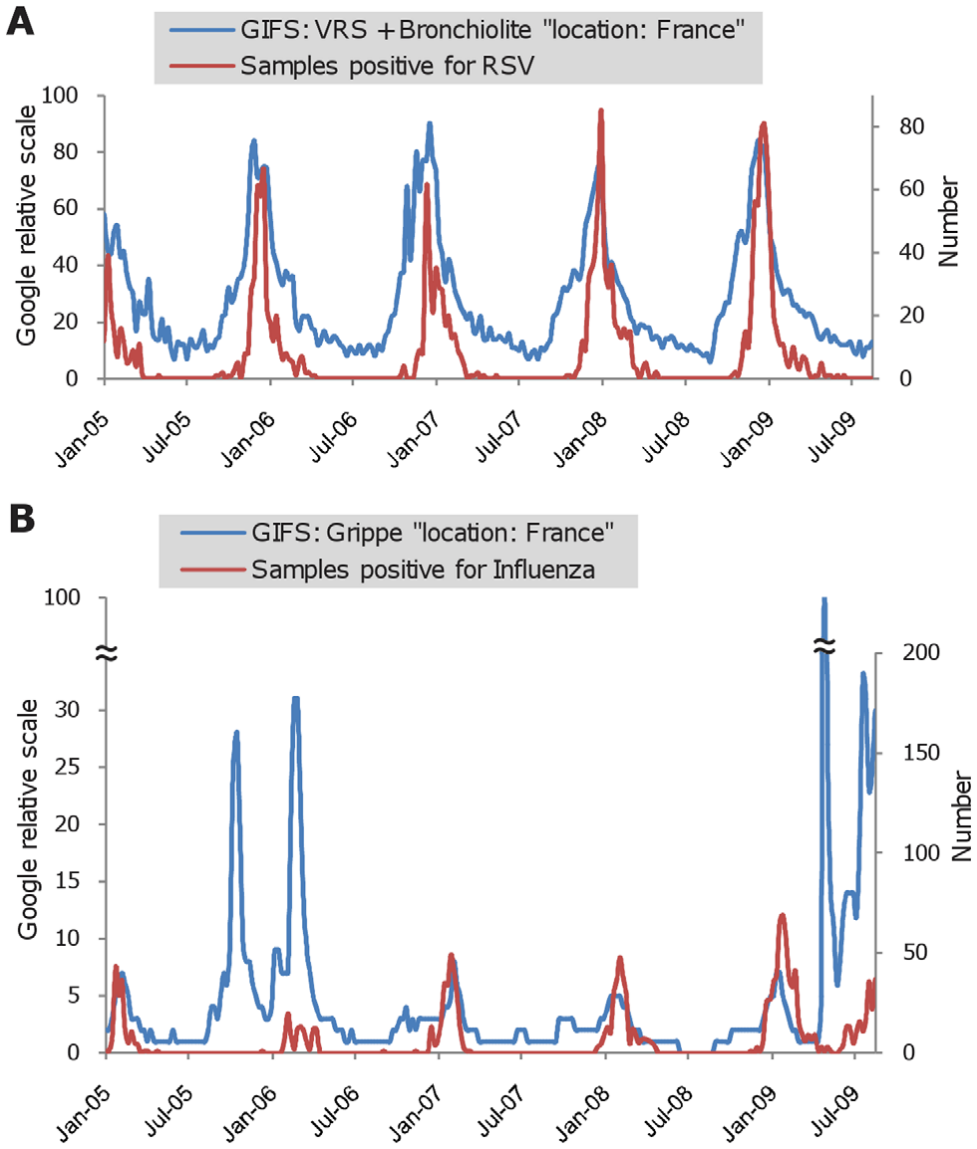
Comparison of Google enquiries in France with data from our Virology laboratory. Figure 4A compares Google enquiry data about RSV infections and figure 4B compares data about influenza.

**Figure 5:**
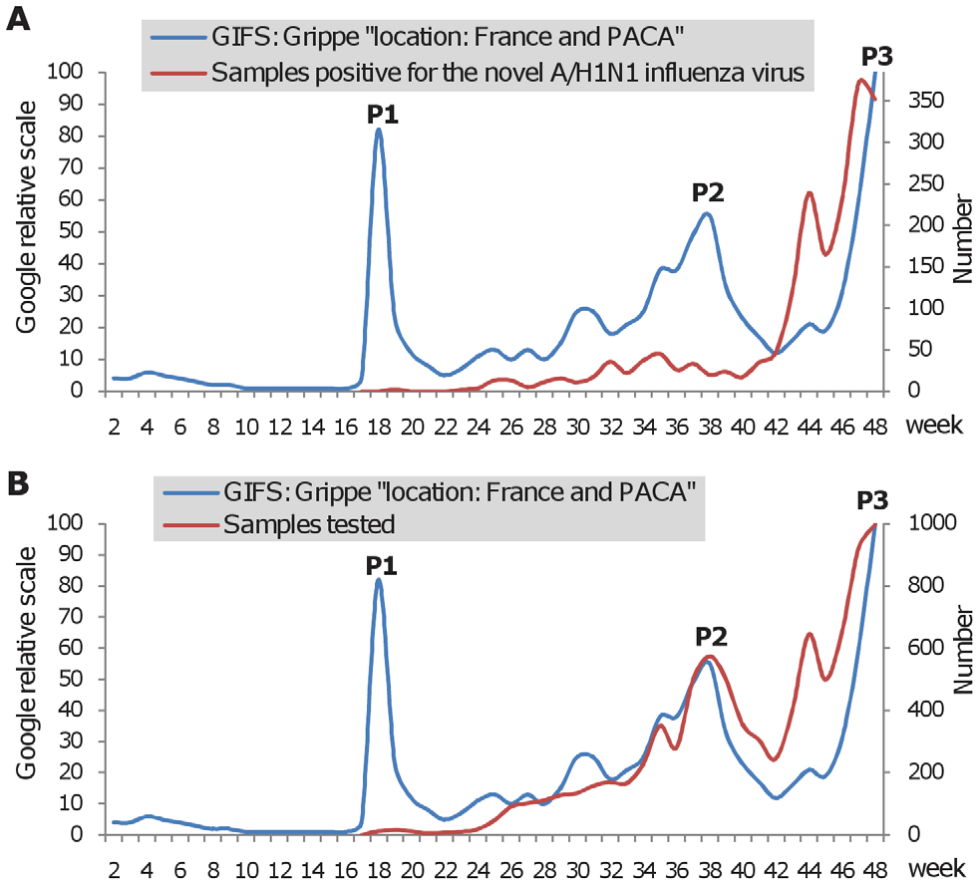
Comparison of Google enquiries in France with data from our Virology laboratory about the novel A/H1N1 Influenza virus. Google enquiries were compared with the number of positive samples (figure 5A) and the number of samples tested (figure 5B). The peaks observed with GIFS were noted P1, P2 and P3.

**Figure 6:**
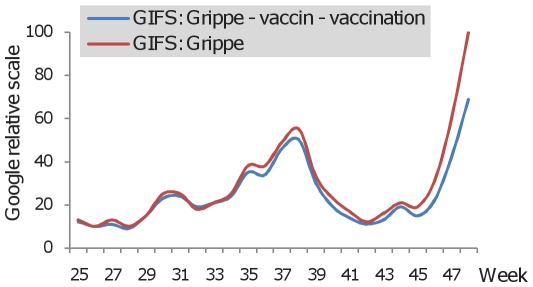
Proportion of Google enquiries in France due to Influenza vaccination.

Based on the information obtained from GIFS, enquiries concerning RSV in France correlated closely with the incidence data observed in the Marseille Virology laboratory (figure 4A). When the two curves representing Google enquiries on RSV and RSV-positive samples were superimposed they matched closely, indicating that GIFS is a sensitive epidemiological surveillance tool for RSV. A correlation was also observed for seasonal Influenza during the 2004-05, 2006-07, 2007-08 and 2008-09 winter waves (figure 4B). However, in the 2005-06 season, long before the winter wave, Google enquiries peaked despite no influenza cases being reported at this time. This huge peak, five times higher than the normal seasonal peak (2004-05, 2006-07, 2007-08, and 2008-09), coincided with the emergence of the novel A/H5N1 highly pathogenic avian Influenza virus [16], [17]. The seasonal Google enquiry peak, observed during the winter of 2005-06, was much higher than that observed in the preceding and following seasons. In the PACA region, before October 2009, the number of laboratory proved A/H1N1 positive samples remained low (less than 50 per week) (figure 5). The pandemic started at the beginning of October (week 40), associated with a simultaneous increase in the number of tested and positive samples. A preceding wave of samples addressed to our laboratory for testing (most of them being negative for A/H1N1) had been collected from early June (week 23) to early October 2009 (week 41). During this period, Google enquiries (P2, figure 5) correlated much better with the number of tested samples than with the number of A/H1N1 positive samples. The first peak of enquiries (P1, figure 5) was observed in late April 2009, when the World Health Organization officially announced the emergence of the novel A/H1N1 virus [18], reflecting the anxiety of the population following news media reports that A/H1N1 was causing epidemics in the Americas, and as previously observed when A/H5N1 avian influenza was widely reported in the news media. When the winter epidemic wave began in Marseille in the autumn of 2009, the increase of Google enquiries (P3, figure 5) was delayed in relation to the number of A/H1N1 positive samples. GIFS analysis showed that enquiries focused exclusively on “influenza” from week 25 to 44. From week 44, enquiries targeting A/H1N1 vaccination increased progressively to reach 30% of the total on “influenza” during week 48 (figure 6).

### Comparison between source Google, newspaper website articles, and PubMed

We compared the nature of information (pro-, con-, neutral) relating to Influenza vaccination available on Google, PubMed and the French newspaper websites (Table 2). We recognise that our classification as pro, con or neutral could be considered subjective,however, in most cases, the opinion expressed was unambiguous. In our view, the information presented here reflects specific opinion. The information relayed by Pubmed was significantly more in favour of Influenza vaccination than either Google or the French newspaper websites. Amongst the Google search results, 11/30 (36.7%) corresponded to French newspaper websites, as the first, second and fourth groups of opinion (neutral, pro- and con- respectively); the third group was obtained from the official Ministry of Health website (pro-) and a fifth issue was from a blog (con-). Whilst all newspapers claim political independence, in France the political tendency of the editorial line of some newspapers is clearly recognized and acknowledged by the public. Thus, the information provided by the French newspaper websites correlated significantly with their recognised political leanings (essentially socialist or conservative)(Table 2).

**Table 2:** Comparison between source Google, newspaper website articles, and PubMed.

			Opinion about influenza vaccination			*p*-value* versus			
			In favour	Against	Neutral	(1)	(2)	(3)	(4)
**Google (1)**			4 (13.3%)	14 (46.7%)	12 (40%)		–	–	–
**PubMed (2)**			20 (66.7%)	1 (3.3%)	8 (26.7%)	*<0.0001*		–	–
**Newspapers websites**	Total		56 (12.4%)	94 (20.8%)	303 (66.9%)	*0.32*	*<0.0001*		
	Socialist tendency (3)	Total	21 (9.7%)	60 (27.8%)	135 (62.5%)	*1.00*	*<0.0001*		–
		Libération	7 (8.9%)	29 (36.7%)	43 (54.4%)				*0.004*
		Le Monde	12 (11.2%)	18 (16.8%)	77 (72.0%)				*0.45*
		L'Humanité	2 (9.1%)	9 (40.9%)	11 (50.0%)				*0.092*
		Marianne2	0 (0.0%)	4 (50.0%)	4 (50.0%)				*0.14*
	Conservative tendency (4)	Total	35 (14.8%)	34 (14.4%)	168 (70.9%)	*0.06*	*<0.001*	*0.0032*	
		Le Point	21 (15.8%)	20 (15.0%)	92 (69.2%)			*0.01*	
		L'Express	7 (16.3%)	7 (16.3%)	29 (67.4%)			*0.13*	
		Le Figaro	4 (22.2%)	3 (16.7%)	11 (61.1%)			*0.19*	
		La Tribune	3 (7.0%)	4 (9.3%)	36 (83.7%)			*0.60*	

*: Chi square test: against versus in favour.

### Trust survey: The public perception of trustworthiness of employees amongst 20 different professions in France

Since our study is concerned with the response of different groups of the general population, to medical recommendations and information delivered via different types of media by several occupational categories (including scientists, physicians, and politicians), we used two trust surveys to assess the level of trust of the French people toward those professions. The results were unambiguous. The first survey (21) found that 89% of the French people questioned claimed that they trusted their doctors whilst only 8% claimed to trust politicians. However, amongst the 20 professions listed in the questionnaire, doctors ranked only fifth behind firefighters (95%), nurses (92%), pharmacists (91%) and airline pilots (88%). Not surprisingly, politicians were placed in 20th position immediately behind car salesmen (15%). The second survey [15] found that scientists and general practitioners were considered to have the most prestigious occupations amongst the 25 professions (ranks 1 and 2, with 58% and 48%, respectively) and the most useful (ranks 3 and 1 with 74% and 79%, respectively) whereas deputies (French members of parliament) ranked 15^th^ (20%) for prestige, and 23^rd^ (24%) for utility.

## Discussion

Prior to the 2009 outbreak of influenza in France, the government sanctioned an order for 94 million vaccine doses as the major countermeasure against the A/H1N1 pandemic strain. It was believed that by devising an appropriate programme and installing the medical infrastructure for immunization, plus governmental advice to the general public, this timely and effective immunization programme would significantly protect France from the major impact of the epidemic. However, the population surprisingly proved to be reluctant to be immunized. Therefore in an attempt to understand what went wrong, we have analysed the major factors that may have contributed to this poor response by the public to the immunization programme. Our results suggest that the reluctance of the general public to be immunized against A/H1N1 influenza virus may have been partly influenced by the sources of information and advice accessed within the French community.

Our analysis of the response to the vaccination programme in the LCPH showed clearly that a relatively high proportion of medically qualified personnel (65%) chose to have the A/H1N1 influenza vaccine whereas the proportion of paramedics, that chose to be immunized, was much lower (22%). A previous study had shown a similar pattern of behaviour during the pre-pandemic H5N1 vaccination programme [19] and this was also the case in a recent study that involved A/H1N1 vaccination among HCWs in Greece [20]. In order to access immunization at the early stages of the immunization programme, paramedics had to attend OMUs, and although they were advised to do this, they received no official information explaining the need for or the benefits of immunization. The resulting compliance was low. Subsequently a local initiative organized by trained medical staff, provided the necessary information and this resulted in a much higher proportion of HCWs being immunized. Similarly, a randomized trial showed that for the seasonal influenza vaccine, MVF significantly increased the vaccination coverage among HCWs in the United-States [21]. Additionally as we showed above, events affecting HCWs directly such as admission to an intensive care unit, modified the perception of the pandemic, in the minds of the HCWs and increased the likelihood of their decision to be vaccinated.

In our study more than 95% of the homeless people included had heard about the pandemic flu vaccine supporting the concept that they have access to the mass media, most likely exchanging the information via group conversation. The wide distribution of free newspapers in France may also contribute to this information source. The compliance observed in homeless persons was relatively high (47.2%), when compared with the poor response to immunization of the general public (3.5% and less than 10% on November 24^th^ 2009 and January 24th 2010 of the eligible population)[9], [22]. This could be partly influenced by the fact that homeless individuals were offered appropriate information by medical teams before taking the decision whether or not to be immunized. Other factors could also explain this higher take-up of the vaccine, for example their relatively precarious living conditions might increase their willingness to accept health care.

We analyzed the information from the internet concerning A/H1N1 epidemicity and the arguments for and against vaccination, because we believed that it may have influenced opinion amongst the general population. Our internet search was performed only in Google, because this is the most popular search engine in France (http://www.indicateur.com/barometre/default_fr.asp). To access Google to obtain the relevant information, we compared the number of enquiries concerning respiratory tract infections with data collected in the Marseille Virology laboratory. Google (i) is often used to search for information on respiratory tract infections; A recent study on A/H1N1 vaccination amongst HCWs in Greece showed that 40.4% of the HCWs questioned had used the internet to obtain information concerning the pandemic [20], and (ii) is a reliable tool to track respiratory tract infections as previously demonstrated [23], [24]. Indeed, in 2005 when the novel A/H5N1 avian Influenza virus which was potentially highly pathogenic for humans, emerged [16], [17], the first big Google enquiry peak was observed. The media emphasised the potential risk of a pandemic associated with this avian influenza virus, increasing general anxiety in the population [25]. This explained the second disproportionate enquiry peak observed during the followed winter seasonal wave of influenza. It had previously been shown that television exposure was one of the major factors that induced the fear of avian flu [25]. Moreover it was proposed that the public consider that infectious diseases most frequently reported to the public by the media, were the most severe [26] and therefore people's anxiety correlated with a negative perception of the disease [27]. During the current pandemic, a major enquiry peak was observed when the World Health Organization announced the emergence of A/H1N1 [18], although it had been featured on the news media for some time. Another major peak of enquiries was observed during the late summer at the start of the academic year. These two peaks coincided with high media exposure of the pandemic. Surprisingly, when the rise in number of A/H1N1 cases commenced in France in mid-October, the expected increase of Google enquiries was delayed. Dramatic increases of Google enquiries demonstrate the sudden interest of people, mainly motivated by anxiety for their own health and that of their families. In addition, the results of the first immunization cohorts were published in the New England Journal of Medicine on September, 12th 2009 [6], [28], 6 weeks before public attention focused on A/H1N1 vaccination through specific Google enquiries. This demonstrates that robust medical data were available but they were consulted almost exclusively by medical staff.

Comparative analyses of the opinions expressed in documents obtained from PubMed (NCBI library) and Google indicate that Google conveyed a high proportion of negative opinion on the advisability of vaccination; their advice was not supported by indisputable scientific evidence. In contrast, PubMed articles presented favourable opinion towards immunization. We recognize that our analysis includes subjective opinion. The reality is clearly extremely complex. However, the New England Journal of Medicine published scientific studies reporting diverse aspects of influenza immunisation (safety, efficacy; cost-effectiveness, morbidity, mortality), 21/22 articles (since 1995) and concluded that immunisation is of benefit regardless of the target population. This is a clear demonstration of the discrepancy between the non-scientifically based information provided via the mass media and the accredited scientific information that is not read by the general public.

In terms of information dissemination, we are living in a rapidly evolving era. We are witnessing a revolution in information retrieval by the general public and in the case of A/H1N1, because much of it conveyed a negative rather than a positive recommendation for immunization, the general French public (at least) appears to a have been influenced by this information. We also believe that official information provided by the scientific community but delivered by Ministry of Health officials was either ignored or simply not heard by a large proportion of the general public. This is corroborated by the results of trust surveys which indicate that politicians are considered much less trustworthy than other important members of society [14]. In contrast, medical practitioners and scientists, amongst others, are perceived as trustworthy and reliable by the general public. The Ministry of Health did not anticipate the impact on the general public of unqualified opinion, expressed on the internet or in the media. With hindsight, it is clear that an entirely new approach needs to be developed in which the medical community, ie practitioners and relevant scientists, should be included in any programme of vaccination and, armed with appropriate information, they should be charged with the responsibility of explaining the importance and significance of vaccination within their local communities. In addition, the role of government, through the Ministry of Health, should be to ensure that the expert advice is delivered widely, by the experts, through the media and the internet, to the general public.

The negative image provided by the mass media could have contributed to the low uptake of the A/H1N1 vaccine in France but other factors could also explain it: in recent studies concerning the attitudes and behaviour towards A/H1N1 vaccine, the main reasons given for not accepting the vaccine were likely to have been: the mild perception of pandemic severity, “I'm not at risk of serious illness”, the fear over vaccine safety, “I'm very sensitive to these vaccines”, and vaccination inefficacy [20], [29], [30], [31]. The discrepancy between the predicted disaster and the relatively mild manifestation of the disease, together with the great deal of discussion concerning vaccine safety, especially the use of Squalene adjuvant, could explain the reluctance of the general population to accept the vaccination. In addition, the eventual willingness to accept the A/H1N1 vaccine was significantly associated with the positive attitude towards seasonal influenza vaccination [20], [29], [30], [32], [33]. Thus, during a potential epidemiological crisis, the measures undertaken to increase vaccination acceptability, might be more effective if they were similar to those taken for seasonal influenza vaccination.

Medical practitioners and scientists, amongst others, are perceived as trustworthy and reliable by the general public. An Australian survey indicated that a proportion of people accepting the A/H1N1 vaccine (11%) would not have been willing to be vaccinated if it took place in a community hall rather than at their GPs, surgery [29]. Specific medical advice and direct (face to face) assistance to the public should be primarily the responsibility of medical practitioners [34]. A French survey based on the acceptance of the A/H1N1, amongst the general population, showed that positive advice by GPs significantly increased the acceptance of A/H1N1 vaccination [32]. Our studies suggest that failure to include GPs in the vaccination campaign could partly explain the failure of the vaccination campaign. Nevertheless, alternative strategies to those developed in France, were developed in some other European countries. In fact, each country developed its own strategy: some purchased sufficient vaccine to immunize the overall population as was done in France, whilst others chose to vaccinate only clinical at risk groups. Moreover, whether or not GPs were involved was not always associated with high rates of immunization. For example, in Germany and Switzerland, GPs were generally associated with the campaign but the final vaccination coverage remained low. Other countries, such as Canada and Sweden, organized the immunization without including the GPs and achieved high rates of immunization.

We need to learn from our mistakes. As in Aesop's tale of The Boy Who Cried Wolf, it is dangerous to predict the likelihood of great catastrophes if they do not subsequently occur. The overly pessimistic predictions of morbidity and mortality on a global scale, resulting from BSE, Smallpox and the Avian H5/N1 flu outbreaks were published in highly-rated scientific journals but were never supported by indisputable scientific evidence. They fuelled the media, causing great concern amongst the public [35], [36], [37], [38], [39]. The failure of these horrible scenarios to materialize generated disbelief and a feeling of “we are being manipulated”. The general French public has therefore become complacent or de-sensitized probably due to the previous perceived failure of the authorities to provide appropriate, helpful and accurate health guidance. Communication efforts should deliver messages based on indisputable scientific evidence. The French strategy to combat the flu pandemic was developed and implemented using a top-down strategy. It was conceived to be a countermeasure to control the disease and also a response to allay the fears of the public generated by the anticipation of the previous avian flu pandemic. However, it failed to convince the general public. Integration of GPs, usually involved in the seasonal flu vaccination campaign, and the first-choice of expert advice and general confidence-building amongst the general population (according to the trust surveys) might have increased vaccine uptake by the general public at a critical time during the first wave of influenza A/H1N1 in France. Fortunately, this first wave of A/H1N1 proved to be relatively mild.
